# Hearing loss assessment in primary and secondary acquired cholesteatoma^☆^^☆☆^

**DOI:** 10.1016/j.bjorl.2014.11.009

**Published:** 2015-09-08

**Authors:** Julia Maria Olsen, Fernando de Andrade Quintanilha Ribeiro, Mariana Mieko Mendes Yasui, Ivan Taylor Ribeiro dos Santos

**Affiliations:** a Faculdade de Saúde Pública, Universidade de São Paulo (USP), São Paulo, SP, Brazil; b Department of Otorhinolaryngology, Faculdade de Ciências Médicas da Santa Casa de São Paulo, São Paulo, SP, Brazil; c Department of Medicine, Faculdade de Ciências Médicas da Santa Casa de São Paulo, São Paulo, SP, Brazil

**Keywords:** Hearing loss, Cholesteatoma, Suppurative otitis media, Audiometry, Perda auditiva, Colesteatoma, Otite média supurativa, Audiometria

## Abstract

**Introduction:**

Acquired middle ear cholesteatoma can be classified as primary or secondary. Although both can result in hearing loss, it is still controversial whether there is an association between the type of cholesteatoma and the degree of hearing loss.

**Objective:**

To analyze the association between hearing loss and the type of acquired cholesteatoma, and the status of the ossicular chain.

**Methods:**

This was a cross-sectional historical cohort study involving patients diagnosed with acquired cholesteatoma who were surgically treated. Air and bone conduction thresholds, air–bone gaps and the status of the ossicular chain were analyzed for both types of cholesteatoma.

**Results:**

Eighty patients aged 5–57 were included in the study. Fifty-one patients had primary cholesteatoma and 29 had secondary cholesteatoma. Both types of cholesteatoma determined greater air–bone gaps at 0.5 kHz. Secondary cholesteatoma determined greater hearing loss in all analyzed frequencies and higher air conduction and air–bone gap means.

**Conclusion:**

There was association between hearing loss and the type of cholesteatoma. Secondary cholesteatoma resulted in greater hearing impairment.

## Resumo

**Introdução:**

O colesteatoma adquirido de orelha média pode ser classificado como primário e secundário. Ambos podem ocasionar perda de audição, mas ainda há controvérsia quanto à relação dos tipos de colesteatoma com a perda auditiva.

**Objetivo:**

Analisar a relação dos tipos de colesteatoma e da erosão da cadeia ossicular com a perda auditiva.

**Método:**

Estudo de coorte histórica com corte transversal, envolvendo pacientes que receberam o diagnóstico de colesteatoma adquirido e foram submetidos à cirurgia otológica. Foram analisados os limiares ósseos, aéreos e a diferença aéreo-óssea, e suas associações com os tipos de colesteatoma e com a presença de erosão na cadeia ossicular.

**Resultados:**

No estudo foram incluídos oitenta pacientes, com idade entre 5 e 57 anos, sendo 51 com colesteatoma primário e 29 com colesteatoma secundário. Ambos os tipos de colesteatoma determinaram maior diferença aéreo-óssea na frequência de 0,5 kHz. O colesteatoma secundário determinou uma perda auditiva maior em todas as frequências analisadas, e maiores médias do limiar aéreo e da diferença aéreo-óssea.

**Conclusão:**

Houve associação entre o tipo de colesteatoma e a perda de audição. O colesteatoma secundário determinou maior comprometimento da audição.

## Introduction

Cholesteatoma is defined as the presence of skin in any air-filled area of the temporal bone, and can be congenital or acquired.^1^ Acquired middle-ear cholesteatoma can be classified according to its origin as primary or secondary.^2^, ^3^, ^4^, ^5^, ^6^ The primary type originates from pars flaccida retraction and the secondary type from the involvement of the pars tensa of the tympanic membrane.^5^, ^7^, ^8^

Studies have shown that the incidence and prevalence of cholesteatoma are much higher in developing countries, highlighting its association with low socioeconomic status, poor hygiene, and delay in seeking health care or poor health services.^9^, ^10^

Cholesteatomas determine mechanical compression of adjacent structures and have cells with hyperproliferative characteristics (cytokeratin 16, KI67, and inflammatory cytokines), which may cause temporal bone and ossicular chain erosion, resulting in hearing loss.^4^, ^11^, ^12^ For this reason and due to the presence of fetid otorrhea, they result in psychosocial damage. As they have a major impact on activities of daily living, they can limit future job opportunities and cause social inclusion difficulties.^9^

There is no consensus in the literature regarding the influence of the type of cholesteatoma on hearing loss, or concerning the frequencies most affected.^11^, ^13^, ^14^, ^15^, ^16^, ^17^

Most studies have shown that there is an association between ossicular chain erosion and magnitude of hearing loss, and that the most affected ossicle is the incus.^12^, ^15^, ^17^

The aim of this study was to analyze the association of primary and secondary acquired cholesteatoma, as well as ossicular chain erosion with hearing loss.

## Methods

The study was approved by the Ethics Committee of the institution under No. 03856512.4.0000.5479.

This was a historical cohort study with cross-sectional design, based on data obtained from medical records of patients treated at the Otorhinolaryngology Outpatient Clinic of a medical teaching institution, between January of 2010 and October of 2013.

*Inclusion criteria*: patients who were diagnosed with acquired cholesteatoma and submitted to otological surgery.

*Exclusion criteria*: patients with other concomitant ear diseases or those who had been previously submitted to otological surgery.

Prior to surgery, all patients underwent pure tone audiometry with determination of air thresholds at 0.25, 0.5, 1–4, 6, and 8 kHz, and bone thresholds at 0.5 and 1–4 kHz. Air and bone thresholds and air–bone gaps were evaluated separately at the frequencies 0.5, 1, 2, and 4 kHz. The means were also evaluated for each patient at those frequencies.

The variables studied were: (a) age and gender; (b) type of cholesteatoma: defined according to clinical history, physical examination data, and intraoperative findings described in the standardized surgical description form, classified as primary cholesteatoma when originating from pars flaccida retraction and secondary when originating from the involvement of pars tensa of the tympanic membrane^8^; (c) presence of ossicular chain erosion, defined by the intraoperative findings, noted as present or absent.

Statistical analysis was performed using Stata™ software, version 11. The association between hearing loss and the assessed variables was performed using Pearson's chi-squared test for categorical variables, and the nonparametric Wilcoxon–Mann–Whitney test for continuous variables, considering a 95% confidence interval and 5% level of significance.

## Results

The present study included 80 patients, 31 females and 49 males, aged between 5 and 57 years (mean age of 26.5 ± 14.75). The left ear was affected in 52.5% of the patients and the right ear, 47.5%. Primary cholesteatoma was found in 51 (63.8%) patients and secondary cholesteatoma in 29 (36.2%) patients. There was no statistically significant difference between the two groups in relation to gender and age (Table 1).

**Table 1:** Distribution of demographic characteristics according to the type of cholesteatoma.

Variable	Primary		Secondary		*p*
	*n*	%	*n*	%	
Gender					0.187^a^
Female	17	33.3	14	48.3	–
Male	34	66.7	15	51.7	–
Mean age	27.1	22.6^b^	25.7	21.8^b^	0.706^c^
Total	51	63.8	29	36.2	

a Chi-squared test.

b Median.

c Non-parametric Wilcoxon–Mann–Whitney test.

As for the hearing impairment at the different frequencies, it was observed that the bone threshold was higher at frequencies 2 and 4 kHz, both in primary and secondary cholesteatoma (Table 2); the air threshold was higher at frequencies 0.5 kHz and 1 kHz in primary cholesteatoma and similar at all frequencies in secondary cholesteatoma (Table 3), whereas the gap was higher at a frequency of 0.5 kHz in both types of cholesteatoma (Table 4).

**Table 2:** Mean bone threshold (in dB) at different frequencies (in kHz) according to the type of cholesteatoma.

Frequency	Primary		Secondary		*p*
	Mean	CI	Mean	CI	
0.5 kHz	7.45	4.90–10.00	11.38	5.59–17.17	0.309
1 kHz	7.05	4.07–10.05	11.96	5.52–18.41	0.172
2 kHz	10.78	7.65–13.92	18.45	11.10–25.80	0.084
4 kHz	10.00	7.16–12.84	16.55	9.40–23.71	0.163

CI, confidence interval; *p*, nonparametric Wilcoxon–Mann–Whitney test.

**Table 3:** Mean air threshold (in dB) at different frequencies (in kHz) according to type of cholesteatoma.

Frequency	Primary		Secondary		*p*
	Mean	CI	Mean	CI	
0.5 kHz	40.29	35.46–45.13	50.52	41.92–59.12	0.029
1 kHz	38.43	33.23–43.62	50.71	42.03–59.39	0.008
2 kHz	33.83	29.12–38.53	50.86	40.51–61.21	0.002
4 kHz	23.40	23.51–39.22	50.34	40.27–60.42	0.038

CI, confidence interval; *p*, nonparametric Wilcoxon–Mann–Whitney test.

**Table 4:** Mean gap (in dB) at different frequencies (in kHz) according to type of cholesteatoma.

Frequency	Primary		Secondary		*p*
	Mean	CI	Mean	CI	
0.5 kHz	32.84	28.50–37.18	39.14	32.81–45.47	0.066
1 kHz	31.37	27.04–35.70	38.75	33.54–43.96	0.017
2 kHz	23.03	18.11–26.63	32.41	27.11–37.71	0.007
4 kHz	28.04	24.21–31.86	33.79	28.28–39.31	0.055

CI, confidence interval; *p*, nonparametric Wilcoxon–Mann–Whitney test.

As for the degree of hearing loss, it was observed that secondary cholesteatoma caused greater loss than primary cholesteatoma at all analyzed frequencies, as well as a larger gap at the frequencies of 1 and 2 kHz (Table 3, Table 4). There was no statistically significant difference in mean bone threshold between primary and secondary cholesteatomas; however, the mean air threshold and the mean gap were higher in patients with secondary cholesteatoma (Table 5).

**Table 5:** Mean hearing thresholds (in dB) according to type of cholesteatoma.

Threshold	Primary		Secondary		*p*
	Mean	CI	Mean	CI	
Bone	8.8	6.4–11.2	14.6	8.5–20.7	0.136
Air	37.6	33.4–41.9	50.6	42.0–59.2	0.006
Gap	28.8	25.2–32.5	36.0	31.3–40.7	0.019

CI, confidence interval; *p*, nonparametric Wilcoxon–Mann–Whitney test.

Only five (6.25%) patients had an intact ossicular chain during surgery, two of them with primary cholesteatoma and three with secondary cholesteatoma. Patients with intact ossicular chain had a mean gap of 26.25 dB (SD ± 7.55 dB) and patients with ossicular chain erosion, 31.77 dB (SD ± 13.54 dB).

## Discussion

The aim of this study was to primarily investigate the association of the types of cholesteatoma with hearing loss and, secondarily, the association between the ossicular chain status and hearing impairment.

The two types of cholesteatoma caused a greater gap at the low frequencies, and 0.5 kHz was affected to a greater degree. Thus, in common with that reported in the literature,^18^, ^19^ this study showed the preservation of the bone threshold and greater involvement of the air threshold at these frequencies.

The difference in hearing loss between both types of cholesteatoma is still controversial in the literature, as the different methodologies make it difficult to compare results.^12^, ^18^, ^19^ In theory, due to its location, secondary cholesteatoma would affect the tense part of the tympanic membrane to a greater extent, the incus and the stapes, causing greater hearing loss. This assumption was confirmed by this study, in which secondary cholesteatoma was associated with more severe hearing loss. Mild hearing loss was found associated with primary cholesteatoma and moderate loss with secondary cholesteatoma, greater findings than those reported in other studies.^17^, ^18^, ^19^

The literature indicates that the degree of hearing loss is not a good predictor of ossicular chain status.^15^, ^16^ However, recent studies have shown that hearing loss is associated with ossicular chain erosion, with the incus being most important ossicle in hearing thresholds.^12^, ^19^ In the present study, the lack of information on the affected ossicle and the small number of cases with an intact ossicular chain at surgery prevented the analysis of the association between hearing loss and the ossicular chain status.

The present study was performed in a public health service, where consultations with specialists, complementary examinations, and surgical treatment still require considerable time.^20^ Thus, the interval between patient symptom onset and their diagnosis and treatment is lengthy. This could explain the high prevalence of ossicular chain erosion and the greater degree of hearing loss we observed.

This study has some limitations. The study includes patients treated at an outpatient clinic rather than a random patient sample, which reduces its ability to be generalized. Additionally, the lack of information on the socioeconomic characteristics of patients and disease duration makes it impossible to investigate possible associations of these variables with hearing loss and the development of explanations for the high prevalence of ossicular chain erosion.

## Conclusions

The type of acquired cholesteatoma resulted in statistically significant differences regarding hearing loss. Secondary cholesteatoma results in greater air threshold impairment and air–bone gap than the primary cholesteatoma. Additionally, both types of cholesteatoma resulted in a greater air–bone gap at the lower frequencies.

## Funding

This study was funded by the PIBIC Scientific Initiation Grant – 10.13039/501100003593CNPq.

## Conflicts of interest

The authors declare no conflicts of interest.
